# Basic knowledge of social hierarchies and physiological profile of reared sea bass *Dicentrarchus labrax* (L.)

**DOI:** 10.1371/journal.pone.0208688

**Published:** 2019-01-09

**Authors:** Pierluigi Carbonara, Maria Dioguardi, Matteo Cammarata, Walter Zupa, Mirella Vazzana, Maria Teresa Spedicato, Giuseppe Lembo

**Affiliations:** 1 COISPA Tecnologia & Ricerca Stazione Sperimentale per lo Studio delle Risorse del Mare, Bari–Torre a Mare, Italy; 2 University of Palermo, Via Archirafi 18, Palermo, Italy; 3 CoNISMa, Piazzale Flaminio, 9 –Roma, Italy; Institut National de la Recherche Agronomique, FRANCE

## Abstract

The effects of social hierarchies (dominant/subordinate individuals), such as aggressiveness, feeding order, and territoriality, are some of the characteristics used for describing fish behaviour. Social hierarchy patterns are still poorly understood in European-reared sea bass (*Dicentrarchus labrax*). In this work, we examine the social interactions among captive fish integrating behavioural and physiological profiles. Groups of three fish with EMG (electromyogram) radio transmitters were monitored for two weeks via video recording. Plasma levels of cortisol, glucose, lactate and lysozyme as well as haematological parameters such as haemoglobin, haematocrit and RBCC (red blood cell count) were measured at the beginning and end of the experiments. Behaviour and muscle activity were monitored daily. The results highlighted that the social hierarchic order was established after one to two days, and it was maintained throughout the experimental period. Dominant and subordinate fish (ß and γ) showed significant differences in muscle activity, hormonal profile (cortisol), aspecific immunity (lysozyme), carbohydrate metabolism (lactate) and behavioural patterns (food order and aggressiveness). This holistic approach helps to provide insights into the physiological status of the subordinate (ß and γ) and dominant individuals. These data have wide implications for aquaculture practice.

## Introduction

The establishment of hierarchy status in fish involves countless interactions among behavioural, endocrine, immunological and energetic reactions to environmental changes [1]. Individual differences in response to challenges are correlated to changes in behaviour [2, 3]. Individual variations in fish has been shown to have implications in a wide range of fields, including behavioural ecology [3], aquaculture [4] and all traits linked to welfare [5], such as health and disease susceptibility, performance traits and the ability to cope with stress [6, 7]. Examples of behavioural parameters that are commonly used as welfare indicators include changes in food-anticipatory behaviour, feed intake and swimming activity [8]. These behaviours are largely reactions to environmental stimuli; therefore, they are a key element in the assessment of fish welfare. Behavioural responses act as a first line in an animal’s defence [9, 10] against adverse environmental changes, predators and social interactions. Such behaviours are often triggered by the same stimuli that activate a response to physiological stress. Fish may exhibit various kinds of behavioural responses to deal with stressful factors, such as the social environment [11, 12, 13]. Dominance relations or hierarchy generally determine social interactions in animals, such as the ability to access resources (e.g. food, water, space and reproductive success) [14]. Fish, as well as other vertebrates, show different behavioural and physiological adaptation strategies that have evolved to cope with challenging situations. Thus, stress can also be viewed as an adaptive function that temporarily allows fish to face environmental changes, safeguarding single specimens and populations [8]. In contrast, chronic stress, which is associated with elevated plasma cortisol levels, can result in a compromised physiological state. For this reason, the mechanisms involved in stress-coping styles in fish have received increasing attention. Social interactions between conspecific fish are, at least for some species, dynamic processes in which subordinate individuals often attempt to become dominant, while the dominant ones seek to maintain their hierarchical status through direct attacks or visual signals [15]. In fish, social defeat is a powerful stressor that can lead to changes in their behaviour and physiology [16, 17, 18]. The dominant position and social status are inextricably linked to the regulation of testosterone and cortisol [19]. Furthermore, it has been demonstrated that cortisol is correlated with the onset of the hierarchy [20].

Among the various responses of fish to stress, changes in swimming pattern have stimulated considerable interest in the scientific community [21, 22, 23, 24]. In fact, swimming, along with other basic functions, is essential for all vital activities of fish, from the search for food and partners to escapes from predators and other dangers. Historically, several tests been used to quantitatively assess swimming activity in fish [25]. Muscle activity, as measured through electromyograms (EMGs) [26, 27, 28, 29], seems to be very responsive. EMGs record bioelectrical voltage changes, which are proportional to the degree and duration of muscle tension as well as to the energetic demand of the individual for swimming and living activities. In recent years, telemetry technology has produced specific EMG tags, which are useful for evaluating fish activity and energy in response to environmental conditions during free swimming in real time [26, 27, 29, 30]. The quantitative monitoring of muscular activity achieved through EMG has already been successfully assessed in relation to different aquaculture conditions, such as starvation and/or feeding periods, transportation activities and rearing densities [5, 27, 28] to assess the impact of such conditions on behavioural modifications [24].

The activation of the hypothalamic–pituitary–interrenal axis (HPI) and the consequent cortisol increase in response to changes in social status are well documented in fish [31]. Cortisol mediates several physiological reactions, such as glucose metabolism, ionic and osmotic regulation and immune response [32]. Most of these responses are species specific [31]. In European sea bass (*Dicentrarchus labrax*), which is one of the most important species for Mediterranean aquaculture [33] but has only recently been domesticated [34], the physiological effects of social status are poorly documented. The relevance of social hierarchic positions in Mediterranean farmed fish has only recently received more attention [6]. Indeed, the time of establishment and duration of the social status are still poorly understood despite the large influence that they exert on the physiology of reared European sea bass [35].

Øverli et al. [2] reviewed various behavioural models reflecting the hierarchical positions of salmonids, sticklebacks and a large number of tropical fish. Social interaction and hierarchical position were demonstrated to delineate a physiological state of the fish, starting from the concentrations of glucocorticoids (the principal hormones involved in the stress response and the ultimate product of HPI axis activation) [2, 36, 31]. The assessment of the physiological state of fish is complex, as countless interactions exist among behavioural, endocrine, immunological and energetic reactions to environmental changes. In addition, individual differences in behavioural and physiological responses are likely to indicate varying adaptations to different types of environments [37].

Di-Poï et al. [38] did not find a well-defined hierarchy relationship in juvenile European sea bass, but the difficulty in drawing conclusions regarding the presence of a hierarchy status could also be partly attributed to the limited understanding of the hierarchical relationship of adults [38]. Although European sea bass has great plasticity in terms of adaptation to captive conditions [35, 36], hierarchic status was demonstrated to be one of the most important drivers of the physiological state of this fish [35, 36, 39]. Moreover, studies on behaviour and copy style in European sea bass have demonstrated some differences between wild and reared fish [35] linked to their behavioural profiles [34, 36].

In light of this background, the aim of this study was to evaluate, for the first time, the establishment of a social hierarchy in adult sea bass (*Dicentrarchus labrax)*, applying a holistic approach to better elucidate the relationships between the behavioural and physiological state of the fish. Several indices were considered, such as haematological and plasma parameters, muscle activity and behaviour. Considering the pivotal role of cortisol in glucose metabolism, ionic and osmotic regulation and the immune system, we also monitored, in addition plasma concentration, glucose, lactate, erythrocyte concentration, haemoglobin, haematocrit and lysozyme. Hence, the effects of the establishment of a social hierarchy on physiological condition were finally assessed using a battery of haematological and physiological indicators in a multi-indicators framework. Indeed, the benefits of using different indicators related to diverse aspects of physiological state are widely recognised [40]. Although this study was conducted with a small group following a mechanistic approach, the implications of the results may be relevant to interpreting the physiological reaction of fish in large groups, as social status has a great influence on the density effects, resource availability, distribution mode in the space and rearing method [35, 37, 38, 39].

## Materials and methods

Adult sea bass (*Dicentrarchus labrax*) were obtained from a commercial hatchery. The experiment was conducted with three European sea bass specimens in triplicate, with fish randomly selected from a batch of 20 fish reared in a 3.5 m^3^ fiberglass tank. In this tank, the oxygen concentration was maintained at overall saturation levels through an automatic system that pumped oxygen when the concentration was reduced to the threshold limit value of 5 mg L^-1^. The water temperature and salinity were kept constant at 18°C and 34%, respectively. The photoperiod was natural, as the fish were reared outdoors. The fish were fed 1% of the total biomass using a commercial feed. All fish (nine specimens with total length [TL] ranging from 35.6 to 39.9 cm; body weight [BW] ranging from 442.3 to 633.4 g) used in the experiment were fluent males (sperm flowing during the manipulation).

The three randomly selected specimens of each replicate were first fasted for 24 hours and then surgically implanted with EMG radio transmitters (Lotek Wireless) following the procedure described by Lembo and colleagues [29]. The specimens were left in the tank to recover from surgical implantation along with the original batch of sea bass in order to avoid the occurrence of hierarchy establishment among the selected specimens.

After the fish completely recovered from the surgical implantation (about five days), they were transferred contemporarily to a 1-m^3^ transparent glass tank (Time 0), which is useful for observing fish behavioural changes. The environmental conditions in the glass tank were kept constant for the entire duration of the experiment (15 days) and were the same as in the batch tank: water temperature of 18°C (± 0.5°C), photoperiod synchronised with external conditions and water recirculation of 10 L min^-1^.

The changing voltage in the red muscle activity was recorded, processed and transmitted to a Lotek Wireless receiver every five seconds as an entire adimensional number, ranging from 0 to 50 [26], which represented the level of fish activity. The EMG radio signals were collected for one hour every day for 15 days. At the same time, the behaviour of each group was observed, and the images were recorded with a digital camera. The EMG data and images were always recorded at the same time in the middle of the light duration to avoid the influence of the sunset and sunrise on the behaviour and muscular activity of the fish. To identify the three individuals in each group, fish were marked with different light-coloured labels. The social status of each specimen was inferred through observation of the fishes’ individual behaviours [41]. Then, each individual was classified, following McCarthy and colleagues [42], as dominant, subordinate ß or subordinate γ. The dominant social status is correlated to higher aggressiveness and preferential access to food [43]. To this end, the number of aggressive acts (A+), defined as a bite or a quick approach without biting that led to the displacement of the subordinate [44], together with the feeding order (FO) were considered. The percentage of attacks was calculated between two individuals (e.g. dominant vs. subordinate ß) and was computed with the following daily mean of attacks formula:

$${A+}_{i\to j}=\frac{\sum_{1}^{k}\frac{n_{i\to j}}{n_{i\to j}+n_{j\to i}}∙100}{k};$$

where k represents the number of experimental days (15 days), and n is the number of attacks observed in 60 minutes of the i specimens against the j specimens. The same calculation was performed for the measurement of preferential access to food.

Before the transfer into the experimental tank (glass tank) (t = 0) and at the end of the experiment, the fish were individually caught and immediately anaesthetised in a 50-L tank using a 30 mg L ^-1^ clove-oil solution [45]. A blood sample of 0.5 mL was collected with a heparinised syringe (needle 23G) from the first branchial arch. Part of the blood was utilised whole (5 μL stored at -20°C for the haemoglobin determination, 5 μL diluted in 1 mL of Hendrick’s solution for the erythrocyte counts and 15 μL to fill three micro haematocrit tubes), and the remaining part was centrifuged at 2000 g for 5 min in order to obtain the plasma, which was stored at -80°C. Then, the following haematological and plasmatic parameters were measured at the beginning (t = 0) and end (after 15 days) of the experiment: haematocrit (Hct), red blood cell count (RBCC), haemoglobin (Hb), plasma cortisol, glucose, lactate, and lysozyme concentration. Haematocrit (per cent packed cell volume, Hct) was determined using a heparinised micro haematocrit tube filled directly from the syringe needle that was centrifuged (15000 g for 3 min) and read immediately after. Hct values were expressed as the percentage of red blood cells to whole blood volume. The red blood cells were counted in a Burker counting chamber under a light microscope (Nikon 400E; Chiyoda, Tokyo, Japan) at 40x magnification, and the final concentration was expressed as the number of cells*10^6^ mm^-3^. Haemoglobin concentration (Hb) was determined through the cyanmethaemoglobin method using a commercial kit (Sigma, St. Louis, MO, USA) and expressed as g dL^-1^. Plasma cortisol was measured using a commercial (InterMedical-Italy) ELISA (enzyme-linked immunosorbent assay) kit for microplate readers (k = 450 nm). In the ELISA kit for cortisol, each 96 well is pre-coated with an antigen. Samples are added to the appropriate microplate wells with antibodies specific for cortisol. The competitive inhibition reaction is launched between pre-coated cortisol and cortisol in the samples. A substrate solution is added to the wells, and the colour develops according to an inverse relationship with the cortisol concentration in the sample. The cross-reactivity between fish cortisol and all the analogues is < 5%, and the minimum detectable dose of fish cortisol is 0.0023 ng mL^-1^. Plasma glucose and lactate concentrations were determined using a commercial kit (Sentinel, Italy) based on the enzymatic colorimetric Trinder reaction (GOD/PAP for glucose and PAP for lactate); both were expressed as mg L^-1^. The plasma lysozyme concentration, which is considered to be an indicator of the functionality of the aspecific immune system, was measured using a turbidimetric assay modified for a microplate reader [46]. The results were expressed as μg mL^-1^ HEWL (hen egg white lysozyme).

### Statistical analysis

Fish body lengths were tested with the Kruskal–Wallis non-parametric test to highlight statistical differences among the three replicates. The behavioural parameters (mean percentage of aggressive acts and preferential access to food) collected during the entire experiment were compared statistically using the Kruskal–Wallis test (p < 0.001) to define the dominant, subordinate ß or subordinate γ specimens for each replicate. The daily means of total aggressive acts by behavioural groups (dominant, subordinate ß and subordinate γ) was analysed using Spearman’s rho coefficient ρ.

Moreover, to exclude a ‘tank effect’, the means of the haematological parameters (Hct, RBCC, Hb, plasma cortisol, glucose lactate and lysozyme) from each replicate were compared (Kruskal–Wallis test) at the 0 time. Because the differences were not significant, all haematological parameters from the three replicates were pooled together, and the averages were statistically compared using the Kruskal–Wallis test within and among the groups (i.e., dominant, subordinate ß and subordinate γ), followed by the post-hoc test.

Likewise, EMG tag data from each replicate were statistically compared (ANOVA) to exclude a ‘tank effect’. Because the differences among the replicates were not significant, the EMG tag data of the dominant, subordinate ß and subordinate γ fish groups were pooled together. The fish muscle activity was averaged and compared statistically among behavioural groups using an ANOVA. Mean daily activity values were also tested in each group for temporal trends using Spearman’s rho coefficient (ρ).

### Ethical considerations

This study was carried out in strict accordance with Directive 2010/63/EU of the European Parliament and the Council of 22 September 2010 on the protection of animals used for scientific purposes. The protocol was approved by the Committee on the Ethics of Animal Experiments of COISPA. All fish manipulations (morphometric measurements, blood samples, surgical implantations) were performed on fish that were completely anaesthetised (stage 4: loss of reflex activity and no reaction to strong external stimuli, as reported by Iversen and colleagues [47]) with a 30 mg L^-1^ clove oil solution [45] to minimise pain and discomfort and to prevent increases in stress [47]. The survival rate after the manipulations (morphometric measurements, blood sample and surgical implantations) was 100%, and all efforts were made to minimise suffering.

## Results

### Behavioural traits

At the start of the experiment, the average TL and BW (± standard deviation) among the three replicates (1° replicate: 37.7 ± 1.8 cm and 531 ± 80.7 g, 2° replicate: 37.4 ± 1.9 cm and 517 ± 91.3 g; 3° replicate: 37.8 ± 2.2 cm and 530 ± 92.8 g) and behavioural groups (Dominant: 37.5 ± 2.1 cm and 523 ± 89 g; Subordinate ß: 37.6 ± 2.2 cm and 523 ± 99 g; Subordinate γ: 36.3 cm ± 1.7–532 g ± 76.2) showed no significant differences (p > 0.05, Kruskal–Wallis test).

The experimental design involved the observation of three European sea bass specimens in triplicate, and the individuals were placed in a tank simultaneously. As shown in Table 1, the percentage of aggressive acts (A+) and preferential access to food (FO) made it possible to classify (p < 0.001, Kruskal–Wallis test) the fish as either dominant or subordinates ß and γ in each group.

**Table 1:** Mean percentage (±SD) of aggressive acts (A+) and preferential access to food (FO). Dom = Dominant; Sub (ß) = subordinate ß; Sub (γ) = subordinate γ.

	Aggressive acts (A+) Mean (%) ± SD	Preferential food accession (FO) Mean (%) ± SD
**Dom *vs*. Sub (ß)**	95 ± 3%	100 ± 0%
**Dom *vs*. Sub (γ)**	96 ± 4%	100 ± 0%
**Sub (ß) *vs*. Sub (γ)**	79 ± 4%	60 ± 1%

**Fig 1:**
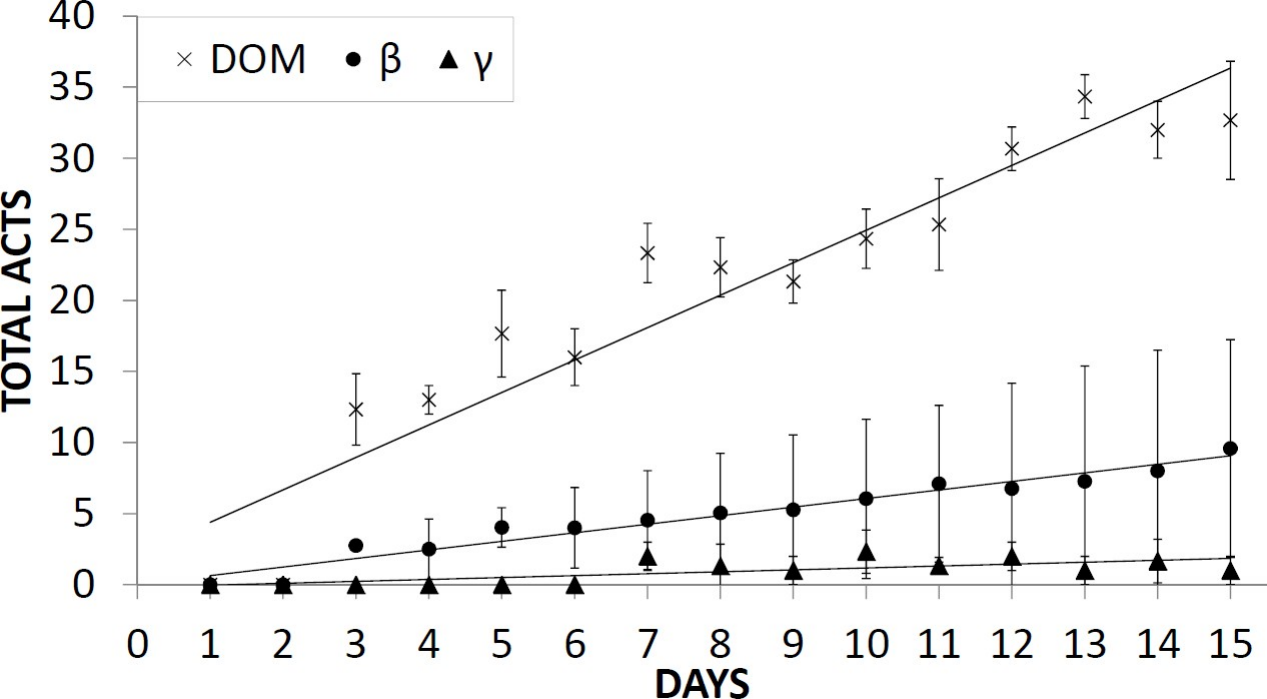
Daily mean (± standard deviation) of total attacks by behavioural groups.

The hierarchy was established within two days, and this characteristic social status remained unchanged throughout the experimental period. Indeed, with regard to the mean daily number of attacks in the first two days, none of fish attacked the others. From the third day onward, the number of attacks by the dominant fish against the subordinates showed a significant increasing trend (ρ = 0.971, p < 0.05). Likewise, the number of attacks by subordinate ß on subordinate γ showed a significant increasing trend (ρ = 0.956, p < 0.05), although the number of attacks by the dominant fish was about three times higher in comparison to the subordinate ß (Fig 1). The number of attacks by subordinate γ was negligible and did not show a significant trend (ρ = 0.551, p > 0.05).

### Haematological and serological parameters

The mean values of haematological parameters (Hct, RBCC, Hb, plasma cortisol, glucose lactate and lysozyme) at the beginning of the experiment did not differ significantly (p > 0.05, Kruskal–Wallis test) among the replicates; therefore, they were pooled together. All the results referring to haematological parameters are reported in Table 2

**Table 2:** Mean values of the haematological and serological parameters for the three groups of fish at the beginning and at the end of the experiment. Statistical differences were tested.

Parameters	Beginning			End		
	Dom	ß	γ	Dom	ß	γ
**HCT** **(%)**	31.26 ± 8.88 * +	29.1 ± 5.88 * #	28.82 ± 8.73 c # +	19.54 ± 3.6 * +	24.47 ± 5.71 b * #	27.43 ± 1.85 c # +
**RBCC** **(10**^**6**^ **cells mm**^**-3**^**)**	2.79 ± 0.47 * +	2.93 ± 3.14 * #	2.73 ± 0.38 c # +	2.11 ± 0.33 * +	2.44 ± 0.56 * #	2.85 ± 0.17 c # +
**Hb** **(g dL**^**-1**^**)**	10.77 ± 2.3 a * +	9.35 ± 1.12 b * #	10.99 ± 0.56 # +	8.28 ± 0.42 a * +	9.67 ± 2.12 b * #	8.39 ± 1.06 # +
**Cortisol** **(ng mL**^**-1**^**)**	84.41 ± 26.79 * +	100.05 ± 13.94 * #	94.27 ± 29.79 # +	47.3 ± 4.92	249.43 ± 32.15	511.87 ± 15.36
**Glucose** **(mg dL**^**-1**^**)**	96.49 ± 37.59 a * +	81.7 ± 28.45 b * #	94.21 ± 34.35 c # +	73 ± 12.48 a * +	80.94 ± 16.98 b * #	95.86 ± 15.19 c # +
**Lactate** **(mg dL**^**-1**^**)**	21.41 ± 4.87 * +	21.93 ± 5.02 b * #	21.8 ± 3.31 c # +	10.63 ± 2.7	20.52 ± 2.7 b #	25.44 ± 3.59
**Lysozyme** **(μg mL**^**-1**^ **HEWL)**	10.46 ± 0.95 * +	10.28 ± 1.36 b * #	9.52 ± 1.93 c # +	12.39 ± 0.49	9.38 ± 2.58 b * #	8.24 ± 0.64 c #

Symbols a, b and c denote a non-significant difference (P < 0.05) within each group (Dom, ß, γ) between the beginning and the end of the experiment, while the symbols *, # and + denote a non-significant difference (P < 0.05) among the groups (Dom, ß, γ) at the two sampling points.

The Hct and RBCC values were significantly lower at the end of the experiment in the dominant group (p < 0.05, Kruskal–Wallis tests); similarly, Hb values were significantly lower in the subordinate γ group (Table 2).

The cortisol levels were not significantly different among the three groups of fish at the beginning of the experiment. However, the cortisol levels were significantly lower in the dominant group and significantly higher in subordinate groups ß and γ (p < 0.05, Kruskal–Wallis test) at the end of the experiment (Table 2).

No significant differences were found in the glucose values between the beginning and end of the experiment or among the three groups. Lactate levels decreased and lysozyme increased, both significantly, in the dominant fish group (p < 0.05, Kruskal–Wallis test) at the end of the experiment (Table 2).

### EMG and muscular activity

**Fig 2:**
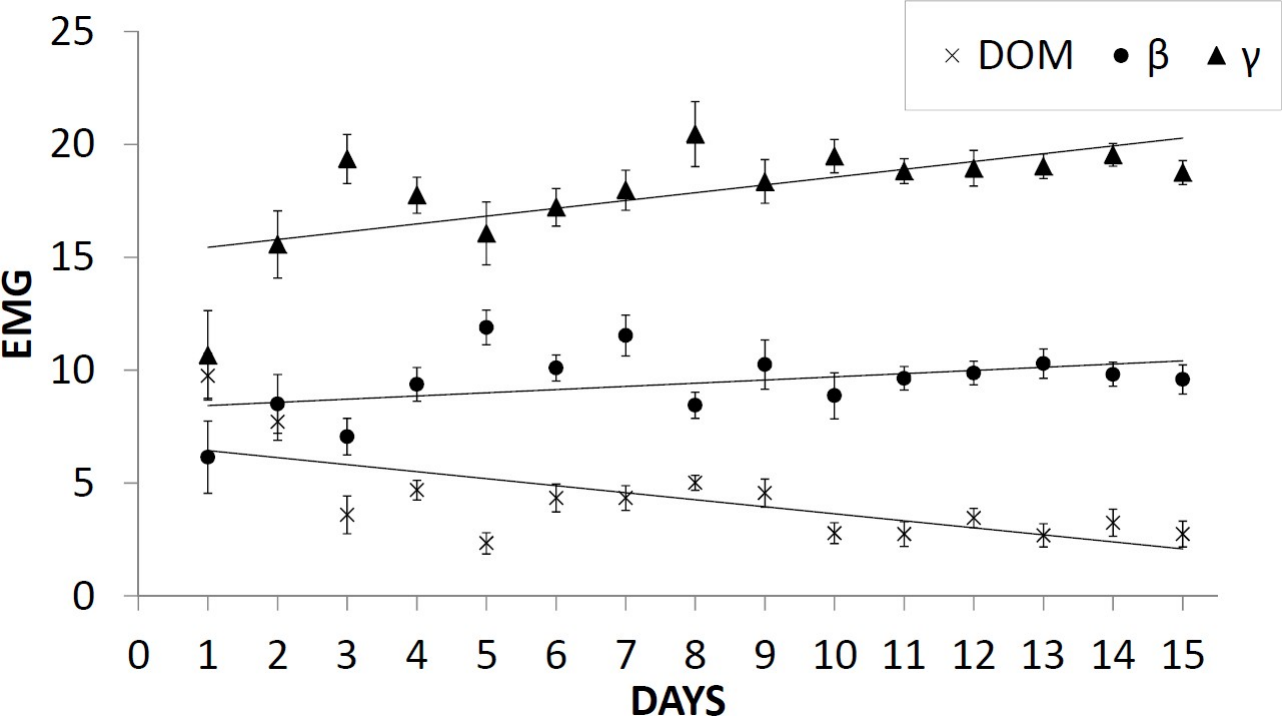
Daily mean swimming activity values (± standard deviation) of the three fish groups.

**Fig 3:**
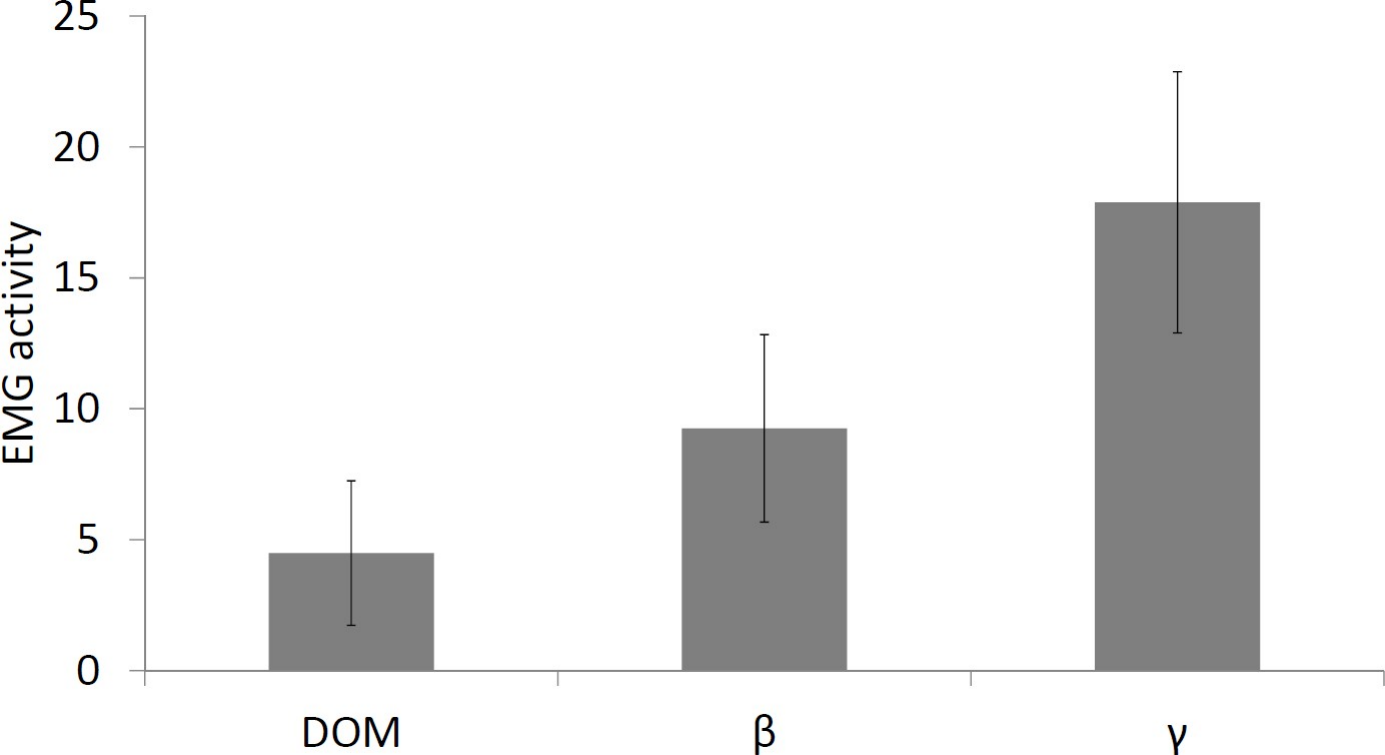
Mean swimming activity values (± standard deviation) of the behavioural groups. All groups show significant differences from each other (p < 0.05).

The level of muscle activity, measured by EMG at the beginning of the experiment, showed no significant differences among the groups (dominant: 9.75 ± 0.995; subordinate ß: 6.15 ± 1.602; subordinate γ: 10.667 ± 1.981; ANOVA, p > 0.05) (Fig 2). However, differences were evident when comparing the mean EMG values of the behavioural groups (dominant: 4.49 ± 2.76; subordinate ß: 9.25 ± 3.58; subordinate γ: 17.88 ± 4.98; ANOVA, p < 0.05) (Fig 3).

The EMG trend of the dominant group showed a significant reduction in the daily mean activity (Spearman’s rho coefficient ρ = -0.632, p = 0.014) during the experiment, while subordinate group ß and subordinate group γ showed, respectively, a non-significant increase in daily mean activity (ρ = 0.386, p = 0.157) and a significant increase in daily mean activity (ρ = 0.618, p = 0.016) throughout the experimental period.

## Discussion

In this study, the hierarchical positions in European sea bass were analysed using a holistic approach, which included examination of haematological and plasma parameters, muscle activity and behaviour. The results highlighted that the hierarchic position is established in adult European sea bass specimens in only a few days (1–2 days) and remains constant for at least for two weeks (experimental duration). The time of establishment of hierarchy seems species specific and is also linked to the experimental conditions [16; 41]. Indeed, there is evidence, as in others species such as the sea bream, that the hierarchy between individuals is established relatively soon, after about one hour of interaction, and it remains unchanged for up to six months of interaction [6]. In this study, we estimated an interval of two days before the hierarchy was established among adult sea bass. The longer time needed to establish hierarchical relationships of *Dicentrarchus labrax* compared to *Sparus aurata* could be due to the different social behaviours of the two species in addition to the different experimental condition. Indeed, studies on wild European sea bass have highlighted gregarious behaviour in juvenile individuals, which is gradually replaced by solitary behaviour in adults [48, 49]. Moreover, in the juvenile phase, no dominance–subordination relationships have been observed in sea bass [50, 51, 52, 53]. Conversely, in other species, such as gilthead sea bream, dominance–subordination relationships have been observed in both the juvenile and adult stages [6]. In our experiment, the influence of handling, as a potential source of stress in terms of time elapsing to the establishment of hierarchies was considered to be very limited, given that European sea bass are able to absorb the handling stress in 3–4 hours [54].

In the present study the dominant sea bass individuals showed a more aggressive attitude and had priority access to food compared to the subordinates (see Table 1), which showed behavioural inhibition, such as suppressed aggression and reduced feed intake [55, 56, 57, 58]. Although dominant individuals can monopolise food, the appetite inhibition in subordinate fish is not merely the result of interference and competition from dominants. The anorexia induced by subordination appears to be mediated by specific brain serotonergic neuronal circuits [59]. Moreover, Currie and colleagues [60] demonstrated that, in juvenile rainbow trout, the dominant and subordinate relationships could have consequences for the animals’ physiological status.

Behaviour affects glucocorticoid levels [61, 62, 63], and, in many cases, when changes in behaviour and glucocorticoid hormones co-occur, causes and effects cannot be easily disentangled. However, subordinate salmonids, as well as individuals of many mammalian species, are often characterised by chronically increased plasma cortisol levels, suppressed aggressive behaviour and reduced food intake [55, 61, 62, 64, 65, 66, 67, 68, 69, 70], which is consistent with the results shown in Table 2.

Indeed, searching for food is an activity that is influenced by cortisol levels [71], suggesting that the interactions between the pathways mediating the stress response and the regulation of food intake in fish are likely to be very complex [72]. Further, as Gregory and Wood [73] pointed out, sustained exposure to elevated cortisol levels may contribute to this behavioural inhibition.

Furthermore, we observed that the dominant individuals tend to occupy, in a decisive manner, part of the tank and maintain more sedentary behaviour, as evidenced by the lower values of lactate at the end of the experiment (Table 2) and the significant decreasing trend of the daily mean EMG activity (Fig 2). The subordinate fish tend to occupy a part of the tank far away from the dominant fish [35] to reduce their risk of suffering an injury in a competition contest [68].

In European sea bass, the establishment of the hierarchy in the adult stage involves not only behavioural patterns, such as aggressiveness and the order of food access, but also a defined physiological profile. In this work, for the first time, behaviour, haematological (Hct, RCCB, and Hb), endocrinology (cortisol), serological (glucose and lactate), immunological (lysozyme) and swimming activity profiles were discussed holistically to obtain a comprehensive picture of the physiological state of the fish.

Significant decreases in some haematological parameters were observed between the beginning and the end of the experiment for the dominant and subordinate ‘γ’ groups. Generally, in European sea bass, fluctuations of haematological profiles are linked to environmental parameters, such as dissolved oxygen [74, 75], water temperature [75] and photoperiod [76]. These parameters were constant during the trial; thus, it is possible that the transfer of the fish into the experimental tank could have caused a temporary increase in some of the haematological parameters. Indeed, acute stress and its consequent release of epinephrine (catecholamine) could be responsible for spleen contraction resulting in an increased release of erythrocytes into the bloodstream [77]. In any case, the variations observed in this study are included in the physiological variations reported in the literature for unstressed and/or untreated European sea bass [5, 46, 75, 76] and thus do not appear to be linked with hierarchical position.

Cortisol plays a pivotal role in the stress response through its action on aerobic and anaerobic metabolism, osmoregulation, carbohydrate metabolism, immunity and appetite [32, 78]. Corticosteroid hormones play a key role in behavioural control, mood, cognition and emotion in a range of vertebrate species [79, 80]. Studies carried out with fish presenting different cortisol levels appear to indicate the role of corticosteroid hormones in the behaviour, cognition and emotions of fish as well as in other vertebrate species [44, 81, 82]. A likely mechanism of the cortisol action in the brain could be its influence on neural turnover [83, 84].

At the beginning of our experiment, the cortisol levels measured in the fish showed no significant differences among the groups and were close to the basal (unstressed) cortisol condition [5, 46, 78]. At the end of the experiment, however, the levels seemed to be correlated to the hierarchical position of each fish. Indeed, the dominant specimens showed significantly lower plasmatic cortisol levels in comparison with the subordinate ß and subordinate γ specimens, and the subordinate ß specimens showed a significantly lower cortisol level in comparison with the subordinate γ specimens. The cortisol levels of fish at the beginning of the experiment and of the dominant specimens were close to the basal (unstressed) cortisol condition [5, 46, 85], while the cortisol levels of the subordinate γ and subordinate ß specimens were comparable, respectively, to the chronic [5, 86, 87] and acute [46, 63, 88] stress conditions.

Although it is not clear whether cortisol itself is the cause of the behavioural changes [17, 89] or if the behavioural changes are due to HPI (hypothalamic–pituitary–interrenal) axis activation [78], some authors argue that the physiological responses to environmental stimuli, such as the HPI axis reactivity, are mostly related to emotional status [89, 90]. The results of our experiment seem to reinforce this last hypothesis. Indeed, fish showing similar initial cortisol levels reached divergent levels at the end of the experimental period as a reaction to the establishment of the hierarchical position; this can be considered an emotional status. In any case, cortisol is the key to explain the change in biochemical and physiological profiles that occurs at the establishment of a hierarchical position. Indeed, according to the results of the present experiment (Table 2), the responses of innate immunity (lysozyme), carbohydrate metabolism (lactate) and muscle activity (EMG) seem to be proportional to the decrease (in dominants) or increase (in subordinates γ and ß) of the cortisol level.

Glucose does not seem to be influenced by the hierarchical position; in fact, no significant variation was observed within or among the groups. The level of plasmatic glucose is comparable with those reported in the literature [45, 63].

Lactate is often used to track the metabolism of carbohydrates, as it is linked to the anaerobic metabolism of carbohydrates, and therefore it could also be used to evaluate white muscle use and the exploitation of anaerobic energy reserves [91]. At the end of the experiment, the subordinates groups (ß and γ) showed significant differences compared to the dominant fish, which presented a significantly lower plasmatic level of lactate along with low muscle activity. The plasmatic lactate concentration is generally used as a secondary response index of a fish’s physiological stress condition because cortisol strongly modulates its release into the bloodstream [77]. Indeed, the lactate levels for the three groups were strongly correlated first with cortisol level and muscle activity (EMG data) and then with hierarchical position.

Lysozyme is used as a general indicator of innate immunity in fish [92]. Acute stress stimuli seem to enhance the non-specific response, whereas chronic stressors seem to depress this kind of immunological reaction [91]. Small fluctuations in cortisol levels do not induce significant variations in immune response, but larger ones [46, 93] and chronic plasmatic cortisol elevation [5, 93] could have negative effects on this haematological parameter [5]. In this experiment, the dominant fish showed a significantly higher plasmatic lysozyme level than the subordinate γ fish. Therefore, in this last group, the depression in innate immunity could be linked to the chronic increase of cortisol levels. While the role of cortisol as a regulator of fish immune systems has not been completely clarified, there is evidence that the type, intensity and duration of the applied stressor may play an important role [32, 94].

EMGs are bioelectrical voltage changes proportional to the degree and duration of muscle tension [95]. These signals are related to the energetic demand and consumption involved in the living activities of free-swimming fish [5, 96]. In many studies, EMGs have been used to describe the physiological state of fish in both confined [5, 27, 28] and wild [97, 98] environments. In this study, EMG data were analysed in relation to hierarchic position. At the beginning of the experiment, when the hierarchic positions were not already defined, the EMG levels did not differ significantly among the groups of fish. However, after a well-defined pattern was established (dominant/subordinates), the muscle activity of the dominant fish was characterised by a significant negative trend, while the muscle activity of the subordinate γ fish showed a significant positive trend. Although the EMG signal is a direct measure of red muscle activity, it has been demonstrated that high EMG signals in European sea bass indicate an intense use of both white and red muscles [29, 96]. Consequently, the living costs of the subordinate fish are higher than those of the dominant fish, both in terms of aerobic and anaerobic metabolism [5, 96], as evidenced by a higher concentration of plasma lactate (Table 2). Hence, it can be argued that because anaerobic metabolism primarily supports the ability to cope with stressful events, subordinate individuals will be less able to face adverse conditions, such as escape, predation and competition. These findings add to the understanding of the behavioural ecology and physiology of sea bass in confined environments. Indeed, the EMG tag makes it possible to monitor free-swimming fish in both confined and wild environments [26], and the general model of swimming activity found in the present study could be used to interpret EMG data from both environments.

Thus, in this study, the hierarchic relationship in reared sea bass was elucidated through the measured indicators (behavioural indicators, muscle activity, haematological and serological parameters), given the fist insights on the time of establishment, duration and physiological traits of dominant and subordinate fish. Further research is needed to examine how the hierarchic relationship works in larger and sex-mixed groups. Although the results of the present study are based on a small group, the basic knowledge regarding the stress consequences of social interaction may contribute to improving the sustainability of the aquaculture industry, including the welfare and performance of farmed fish, starting for example for interactions among isolated broodstock. While the sea bass is a non-territorial fish and the breeding conditions and confined experiment were different from natural ones, the integration between experimental behaviourism and eco-ethological observation has been often been used for phenomenon comprehension.

## Conclusion

The present study shows that hierarchic position is established in adult European sea bass specimens after about two days and remains constant for at least two weeks (experiment duration).

Moreover, the dominant fish exhibit a lower level of cortisol, muscle activity, use of anaerobic metabolism and a higher level of innate immunity activity compared to subordinate fish, which, conversely, show a higher level of cortisol, muscle activity, use of anaerobic metabolism and a lower level of innate immunity. Therefore, it could be concluded that the dominant fish shows a lower ‘living cost’ in comparison to the subordinate ones under the simple experimental conditions of the present study. All this is channelled into greater energy reserves to allow the dominant fish to face stress conditions (i.e. competition, escape, disease resistance, etc.). These findings represent a further step toward better understanding how fish face, with coping strategies or coping styles the different environmental conditions/enrichment found in aquaculture farms [99].

## Supporting information

S1 Table Length (mm) and Weight (g) of European seabass involved in the experiment. (PDF)

S2 Table Haematological and serological parameters (Glucose [mg dL^-1^]; Lactate [mg dL^-1^]; Cortisol [ng mL^-1^]; Lysozyme [μg mL^-1^ HEWL]; HCT [%]; Hb [g dL^-1^]; RBCC [10^6^ cells mm^-3^]), for each fish at the beginning and at the end of the experiment. (PDF)

S3 Table Number of attacks carried out by fish per experiment day. (PDF)

S4 Table Electromyogram (EMG) by hierarchy position of specimens and time (experiment day). (PDF)
