# Supplementary material for: Basic knowledge of social hierarchies and physiological profile of reared sea bass Dicentrarchus labrax (L.)
Source: PLoS One. 2019 Jan 9;14(1):e0208688. doi: 10.1371/journal.pone.0208688 (PMC6326550; doi:10.1371/journal.pone.0208688)
Supplement: S1 Table — (PDF) [file pone.0208688.s001.pdf]

S1

Replicate	Hierarchy	TL (mm)	weight (g)
1	Dom	397	624.0
1	β	373	493.0
1	γ	362	477.0
2	Dom	372	491.0
2	β	356	442.3
2	γ	395	619.0
3	Dom	356	455.0
3	β	399	633.4
