# Supplementary material for: Basic knowledge of social hierarchies and physiological profile of reared sea bass Dicentrarchus labrax (L.)
Source: PLoS One. 2019 Jan 9;14(1):e0208688. doi: 10.1371/journal.pone.0208688 (PMC6326550; doi:10.1371/journal.pone.0208688)
Supplement: S2 Table — (PDF) [file pone.0208688.s002.pdf]

S2

			mg/dL	mg/dL	ng/mL	µg/mL	%	g/dL	cells*10^6/mm^3
Sampling period	Hierarchy	Replicate	Glucose	Lactate	Cortisol	Lysozyme	HCT	Hb	RBCC
Begin	Dom	1	66.192	21.1258	113.2004	11.41026	23.87	8.33	23.87
Begin	Dom	2	138.553	26.423	60.21643	9.512195	28.8	11.06	28.8
Begin	Dom	3	84.73531	16.69177	79.81106	10.45732	41.11282	12.91	41.1128161
Begin	β	1	59.212	22.416	115.6709	11.41026	22.48	8.263564	22.48
Begin	β	2	113.683	26.688	88.86385	10.67073	33.71	10.49346	33.71
Begin	β	3	72.19333	16.69177	95.61948	8.770325	31.11499	9.301397	31.1149932
Begin	γ	1	67.884	21.092	128.1294	11.16667	22.05	11.47705	22.05
Begin	γ	2	133.067	25.404	82.58157	9.989837	25.72	11.11377	25.72
Begin	γ	3	81.68256	18.90731	72.09775	7.398374	38.67654	10.36926	38.67653517
End	Dom	1	87.216	13.751	52.20681	12.01282	17.64	8.04	17.64
End	Dom	2	63.86	9.062	42.36444	12.21341	17.28	8.04	17.28
End	Dom	3	67.93637	9.072723	47.33211	12.94512	23.68774	8.76	23.68774076
End	β	1	62.77	20.849	240.4523	11.69231	21	7.225549	21
End	β	2	96.406	23.043	222.7198	9.857724	31.07	10.73187	31.07
End	β	3	83.62933	17.66922	285.1058	6.595528	21.35395	11.04116	21.3539468
End	γ	1	79.524	21.627	507.1258	7.8	26.57	7.173653	26.57
End	γ	2	109.564	28.75	499.4479	7.953252	29.55	8.872255	29.55
End	γ	3	98.48053	25.9399	529.0496	8.973577	26.16571	9.113772	26.16571087
