# Supplementary material for: Basic knowledge of social hierarchies and physiological profile of reared sea bass Dicentrarchus labrax (L.)
Source: PLoS One. 2019 Jan 9;14(1):e0208688. doi: 10.1371/journal.pone.0208688 (PMC6326550; doi:10.1371/journal.pone.0208688)
Supplement: S3 Table — (PDF) [file pone.0208688.s003.pdf]

S3

Hierarchy	Replicate	number of attacks	experimental day
Dom	1	0	1
Dom	2	0	1
Dom	3	0	1
Dom	1	0	2
Dom	2	0	2
Dom	3	0	2
Dom	1	10	3
Dom	2	12	3
Dom	3	15	3
Dom	1	13	4
Dom	2	14	4
Dom	3	12	4
Dom	1	15	5
Dom	2	17	5
Dom	3	21	5
Dom	1	18	6
Dom	2	14	6
Dom	3	16	6
Dom	1	21	7
Dom	2	24	7
Dom	3	25	7
Dom	1	20	8
Dom	2	24	8
Dom	3	23	8
Dom	1	21	9
Dom	2	23	9
Dom	3	20	9
Dom	1	25	10
Dom	2	26	10
Dom	3	22	10
Dom	1	23	11
Dom	2	24	11
Dom	3	29	11
Dom	1	32	12
Dom	2	31	12
Dom	3	29	12
Dom	1	34	13
Dom	2	33	13
Dom	3	36	13
Dom	1	32	14
Dom	2	34	14
Dom	3	30	14
Dom	1	28	15
Dom	2	34	15
Dom	3	36	15
β	1	0	1

β	2	0	1
β	3	0	1
β	1	0	2
β	2	0	2
β	3	0	2
β	1	4	3
β	2	3	3
β	3	2	3
β	1	6	4
β	2	3	4
β	3	4	4
β	1	6	5
β	2	7	5
β	3	4	5
β	1	7	6
β	2	5	6
β	3	4	6
β	1	7	7
β	2	4	7
β	3	3	7
β	1	6	8
β	2	4	8
β	3	8	8
β	1	5	9
β	2	9	9
β	3	4	9
β	1	7	10
β	2	8	10
β	3	3	10
β	1	4	11
β	2	5	11
β	3	9	11
β	1	7	12
β	2	6	12
β	3	9	12
β	1	4	13
β	2	6	13
β	3	8	13
β	1	10	14
β	2	5	14
β	3	8	14
β	1	9	15
β	2	11	15
β	3	6	15
γ	1	0	1
γ	2	0	1
γ	3	0	1
γ	1	0	2
γ	2	0	2
γ	3	0	2
γ	1	0	3

γ	2	0	3
γ	3	0	3
γ	1	0	4
γ	2	0	4
γ	3	0	4
γ	1	0	5
γ	2	0	5
γ	3	0	5
γ	1	0	6
γ	2	0	6
γ	3	0	6
γ	1	2	7
γ	2	3	7
γ	3	1	7
γ	1	0	8
γ	2	1	8
γ	3	3	8
γ	1	0	9
γ	2	1	9
γ	3	2	9
γ	1	1	10
γ	2	2	10
γ	3	4	10
γ	1	1	11
γ	2	1	11
γ	3	2	11
γ	1	2	12
γ	2	3	12
γ	3	1	12
γ	1	0	13
γ	2	1	13
γ	3	2	13
γ	1	3	14
γ	2	0	14
γ	3	2	14
γ	1	1	15
γ	2	0	15
γ	3	2	15
