# Supplementary material for: Basic knowledge of social hierarchies and physiological profile of reared sea bass Dicentrarchus labrax (L.)
Source: PLoS One. 2019 Jan 9;14(1):e0208688. doi: 10.1371/journal.pone.0208688 (PMC6326550; doi:10.1371/journal.pone.0208688)
Supplement: S4 Table — (PDF) [file pone.0208688.s004.pdf]

S3

Hierarchy	experimental day	EMG mean	sem
DOM	1	9.75796225	0.99551378
DOM	2	7.70855486	0.81707511
DOM	3	3.59508734	0.83420209
DOM	4	4.68918635	0.43246254
DOM	5	2.33745773	0.46343272
DOM	6	4.34588954	0.62038331
DOM	7	4.34211673	0.54751732
DOM	8	5.01641992	0.32979827
DOM	9	4.55421851	0.61896725
DOM	10	2.77813736	0.46189459
DOM	11	2.73750932	0.54618671
DOM	12	3.45263158	0.42110965
DOM	13	2.68322981	0.51199377
DOM	14	3.24403409	0.59527268
DOM	15	2.74535724	0.56888028
β	1	6.15048137	1.60209052
β	2	8.50658095	1.30060732
β	3	7.0578564	0.81006195
β	4	9.37026791	0.75212561
β	5	11.8928396	0.75814997
β	6	10.0943026	0.57756584
β	7	11.5372558	0.90458388
β	8	8.44466636	0.57445296
β	9	10.2456265	1.09196142
β	10	8.8734122	1.01875362
β	11	9.64122013	0.51853084
β	12	9.87586122	0.52431715
β	13	10.2911505	0.65414289
β	14	9.81501715	0.53537706
β	15	9.59270375	0.64289987
γ	1	10.6666667	1.98115787
γ	2	15.5714286	1.48968169
γ	3	19.35646	1.07887204
γ	4	17.7563636	0.79760735
γ	5	16.0561224	1.39064302
γ	6	17.2201258	0.83888058
γ	7	17.9704861	0.88720359
γ	8	20.4560531	1.43883786
γ	9	18.3548	0.96641743
γ	10	19.47852	0.7448439
γ	11	18.8227848	0.55301675
γ	12	18.9432177	0.79294107
γ	13	19.0326679	0.54130985
γ	14	19.545	0.50723899
γ	15	18.754156	0.52998138
